# Mechanisms of activation of innate-like intraepithelial T lymphocytes

**DOI:** 10.1038/s41385-020-0294-6

**Published:** 2020-05-15

**Authors:** Maud Vandereyken, Olivia J. James, Mahima Swamy

**Affiliations:** MRC Protein Phosphorylation and Ubiquitylation Unit, School of Life Sciences, University of Dundee, Dundee, DD1 5EH UK

## Abstract

Intraepithelial T lymphocytes (T-IEL) contain subsets of innate-like T cells that evoke innate and adaptive immune responses to provide rapid protection at epithelial barrier sites. In the intestine, T-IEL express variable T cell antigen receptors (TCR), with unknown antigen specificities. Intriguingly, they also express multiple inhibitory receptors, many of which are normally found on exhausted or antigen-experienced T cells. This pattern suggests that T-IEL are antigen-experienced, yet it is not clear where, and in what context, T-IEL encounter TCR ligands. We review recent evidence indicating TCR antigens for intestinal innate-like T-IEL are found on thymic or intestinal epithelium, driving agonist selection of T-IEL. We explore the contributions of the TCR and various co-stimulatory and co-inhibitory receptors in activating T-IEL effector functions. The balance between inhibitory and activating signals may be key to keeping these highly cytotoxic, rapidly activated cells in check, and key to harnessing their immune surveillance potential.

## Introduction

Intestinal intraepithelial T lymphocytes (T-IEL) constitute the largest T cell compartment in the body, with a ratio of at least 1 T-IEL for every 10 epithelial cells lining the entire human gastrointestinal tract.^1^ T-IEL are commonly classified into two main groups based on their mode of selection in the thymus, and their functional potential: induced, adaptive or conventional T-IEL and natural, innate-like or unconventional T-IEL.^2,3^ Induced T-IEL develop from conventional CD4^+^ or CD8αβ^+^ TCRαβ^+^ T cells that are activated post-thymically in response to peripheral antigens, and then migrate to the gut as memory-type lymphocytes. In the murine small intestine, the majority of the induced T-IEL are TCRαβ CD8αβ^+^, whereas CD4^+^ cells account for only 10% of the total T-IEL population. Natural T-IEL are thought to acquire their phenotype in the thymus upon self-antigen encounter, although this is still debated, and are CD4^−^ and CD8αβ^−^, therefore sometimes referred to as double-negative (DN) T-IEL.^4,5^ These natural T-IEL are also referred to as innate-like due to their ability to be activated by cytokines and other innate ligand-receptor axes, in a TCR-independent manner. The majority of murine innate-like T-IEL are characterized by the expression of CD8αα homodimers, and express either TCRαβ or TCRγδ. These innate-like T-IEL comprise up to 80% of murine T-IEL and share a gene expression profile that is distinct from induced IEL.^3^ Based on this gene expression profile, innate-like T-IEL also exist in humans and represent 5–30% of the T-IEL compartment, although they rarely express CD8αα (Table 1). The human adult IEL compartment is therefore mainly composed of induced TCRαβ CD8αβ (∼80%) and TCRαβ CD4 (∼10%) T-IEL.^6^ It should be noted here that these so-called induced T-IEL are antigen-experienced T cells that also have the capacity to respond in an antigen-independent manner, and exhibit other innate-like characteristics, such as expression of NK receptors^7–9^ (Table 1). Thus the distinction between these two cell types within the epithelium may be blurred, in order to permit IEL to rapidly respond to changes at the epithelium.

**Table 1:** Intestinal human and murine innate-like subset representation, their TCR repertoire and receptor expression profile.

T-IEL type	TCR ligands	Receptors expressed
*Mouse*		
Innate-like TCRγδ CD8αα	Nonclassical MHC; Btnl1/Btnl6 (Vγ7)	NKG2D^a^; CD160^b^; CD100^b^; Ly49E^b^; CD200R^b^; 2B4^b^; JAML^b^; LAG-3^b^; TIGIT^b^; Gp49^b^
Innate-like TCRαβ CD8αα	Classical and nonclassical MHC class I and II (cross-reactive)	NKG2D^a^; CD160^b^; CD100^b^; Ly49 family^b^; CD200R^b^; CD94/NKG2^a,b^; 2B4^b^; JAML^b^; LAG-3^b^; TIGIT^b^; Gp49^b^
Induced TCRαβ CD8αβ or CD4	MHC class I or II	CD160^b^; CD100^b^; CD200R^b^; 2B4^b^; JAML^b^; CTLA-4^b^
*Human*		
Innate-like TCRγδ	Nonclassical MHC; BTNL 3/8 (Vγ4)	NKp46^b^
Innate-like TCRαβ	unknown	unknown
Induced TCRαβ CD8αβ or CD4	MHC class I or II	NKG2D^a^; CD94/NKG2^a,b^; Nkp46^a^

T-IEL subset composition differ from humans to mice. In humans, induced TCRαβ CD8αβ T-IEL are predominant while in mice, the majority of T-IEL is composed of innate-like T-IEL (TCRαβ CD8αα and TCRγδ CD8αα). In addition to their TCR, human and murine T-IEL express numerous activating and inhibitory receptors, suggesting alternative modes of activation.

^a^Expression induced by inflammatory signals or in the context of disease.

^b^Constitutively expressed.

T-IEL are thought to play a critical role in preserving the intestinal barrier integrity by controlling the growth, turnover and repair of the epithelium, while also defending it from infection.^10^ Studies using TCRδ-deficient mice lacking γδ T cells highlighted a critical role of γδ T-IEL in influencing turnover and differentiation of intestinal epithelial cells (IEC)^11^ and in maintaining mucosal homeostasis. This function of T-IEL has been particularly shown to be important in the recovery from colitis-inducing chemicals in mouse models.^12,13^ Conversely, T-IEL are also cytotoxic cells that patrol the epithelial layer to sense and eliminate infected/damaged epithelial cells.^14^ They are activated in response to oral infections with pathogens such as Salmonella,^15,16^ Rotavirus,^17^ and Toxoplasma gondii,^18^ and at least in the context of loss of γδ T-IEL function, they appear to restrict pathogen entry.^15,16,19,20^ Given their constant exposure to the gut microenvironment they must have the capacity to tolerate commensal bacteria while also being able to quickly recognize and fight enteric pathogens. To achieve this, they are kept in an “activated, yet resting” state, ready for action, but tight control of this poised activation state is essential.^2^ If this control is disrupted, T-IEL might potentially exacerbate the intestinal damage found in autoimmune and inflammatory bowel diseases (IBD)^21–25^ such as Crohn’s and coeliac disease. It is therefore of vital importance to understand which signals activate T-IEL cytotoxic responses, and which signals trigger their tissue reparative functions.

**Fig. 1:**
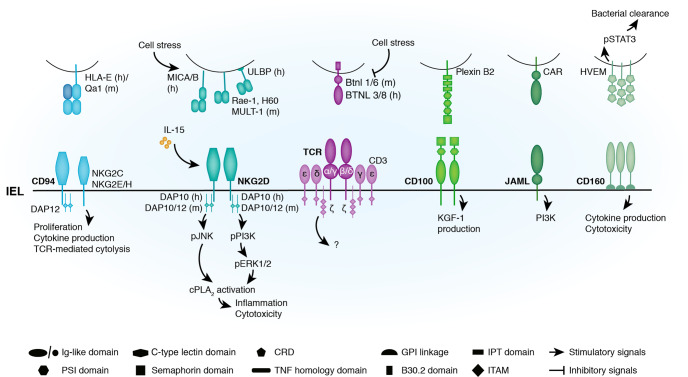
Stimulatory receptors expressed on intestinal intraepithelial T lymphocytes. T-IEL express (co)stimulatory receptors (NKG2D, CD94, CD100, JAML, and CD160) that regulate TCR activation, T-IEL cytotoxicity and their cytokine production. Associated ligands are often found within the intestine, mainly at the surface of epithelial cells. The expression of some receptors and their ligands can be modulated by the microenvironment. For example, NKG2D is upregulated when cells are exposed to high levels of IL-15 and expression of NKG2D ligands and BTNL molecules are modulated by cell stress and inflammation. Although it is still not clear how these (co)stimulatory receptors regulate innate-like T-IEL activation, known signaling events are depicted. T-IEL cytotoxicity triggered by NKG2D activation in coeliac disease depends on PI3K/ERK and JNK-mediated cPLA_2_ activation. Conversely, CD100 engagement on γδ T-IEL is important for wound repair, possibly through the production of KGF-1. In addition to inducing cytokine production and enhancing T-IEL cytotoxicity, receptor-ligand engagement can also trigger signaling in epithelial cells, as evidenced by HVEM engagement on epithelial cells leading to enhanced bacterial clearance in infection. (h) human; (m) mouse; Ig immunoglobulin, CRD cysteine-rich domain, GPI glycosylphosphatidylinositol, PSI domain plexins, semaphoring and integrin domain, IPT domain Ig-like, plexin and transcription factors domain, ITAM immunoreceptor tyrosine-based activation motif, TNF tumor necrosis factor, PI3K Phosphoinositide 3 kinase, pPI3K phosphorylated PI3K, pJNK phosphorylated c-Jun N-terminal Kinase, pERK1/2 phosphorylated Extracellular signal-Regulated Kinase 1/2, cPLA2 cytosolic Phospholipase A2, KGF keratinocyte Growth Factor, pSTAT3 phosphorylated Signal Transducer and Activator of Transcription 3.

**Fig. 2:**
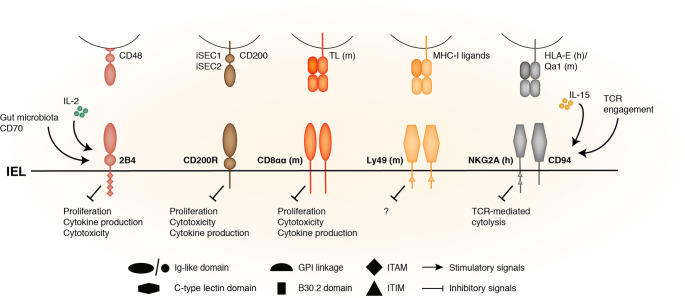
Inhibitory receptors expressed on intestinal intraepithelial T lymphocytes modulate their function. In addition to (co)stimulatory receptors, innate-like T-IEL express inhibitory receptors such as CD8αα, CD200R, 2B4, Ly49, and CD94/NKG2A that regulate TCR activation, their proliferation, their cytotoxicity, and their cytokine production. CD8αα and Ly49 are only expressed by murine T-IEL. 2B4 expression is induced by gut microbiota and CD70 co-stimulation. TCR engagement and IL-15 upregulate CD94 expression. Like (co)stimulatory receptor ligands, ligands for inhibitory receptors are often found within the intestine, mainly at the surface of epithelial cells, suggesting that inhibitory receptor engagement is necessary for keeping T-IEL in an “activated, yet resting” state. Indeed, by preventing aberrant T-IEL proliferation, cytotoxicity and cytokine production, inhibitory signals may maintain T-IEL and gut homeostasis. In line with this, in coeliac disease, the CD94/NKG2A heterodimer expression is selectively downregulated while CD94/NKG2C pair is upregulated, tipping the balance toward effector function of T-IEL. (h) human; (m) mouse; Ig immunoglobulin, GPI glycosylphosphatidylinositol, ITAM immunoreceptor tyrosine-based activation motif, ITIM immunoreceptor tyrosine-based inhibitory motif, TL thymus Leukemia antigen.

Here, we discuss recent evidence indicating that T cell antigen receptors (TCR) ligands for some innate-like T-IEL can be found on epithelial cells and are essential for their selection and maturation. We also review the data addressing whether the TCR is an activating receptor on innate-like T-IEL. In addition to their TCR, both induced and innate-like T-IEL express many unconventional signaling receptors not normally found on naïve T cells, such as NKG2D, 2B4 and CD160, which suggest alternate modes of activation, that may or may not involve the TCR (Fig. 1). Conversely, T-IEL express a slew of co-inhibitory receptors that may be key to keeping these highly cytotoxic, rapidly activated cells in check (Table 1 and Fig. 2). The importance of these receptors on T-IEL are the subject of many new studies, and herein we review them to provide an overview of our understanding of T-IEL activation and regulation. We also draw on comparisons with studies of skin-resident dendritic epidermal T cells (DETCs), as an example of a tissue-resident γδ T-IEL population whose activation has been evaluated in more detail, due to the better tools available to study this innate-like T cell population.

## The T cell antigen receptor

Expression of a TCR is the defining feature of T cells, and its engagement by antigen is considered essential for T cell function. In this section we explore the relevance of the TCR for the selection of innate-like T-IEL and for their functional activation.

## Selection of antigen specificities of intestinal T-IEL TCRs

Both αβ and γδ T-IEL derive their TCRs from recombination-activating genes (RAG)-dependent gene rearrangements that occur in developing precursors in the thymus. Initial studies showed that, while there was some bias in V gene usage, intestinal innate-like αβ T-IEL are essentially oligoclonal populations with diverse TCRs that undergo clonal expansion in the gut.^26,27^ Conversely, the majority of intestinal γδ T-IEL express the Vγ7 gene, although this chain still has considerable junctional diversity, and is paired with diverse Vδ chains.^28–30^ This is in contrast to DETC in the murine skin, where >90% of the cells express the Vγ5Vδ1 TCR. The DETC TCR is selected in the fetal thymus by an immunoglobulin superfamily molecule, the Selection and upkeep of intraepithelial T-cells protein 1 (Skint1).^31^ Although formal proof that Skint1 can bind to the Vγ5Vδ1 TCR is still lacking, modeling studies and in vitro analyses suggest this to be the case,^32^ and it is thought that intrathymic Skint1-TCR interaction drives DETC selection and maturation.

The search for a Skint1-equivalent molecule for the selection and function of intestinal γδ T-IEL lead to the discovery of Butyrophilin-like (Btnl) family of molecules.^33^ Btnl1 is expressed as a heterodimer with Btnl6 on IEC under homeostatic conditions.^34^ Hayday and colleagues recently demonstrated that in the absence of Btnl1, Vγ7^+^ T-IEL were almost absent from the intestinal compartment^35^ (Fig. 1 and Table 1). Critically, they could demonstrate that the Vγ7 TCR is essential for the response to Btnl1/Btnl6, and thus drives the selective expansion and maturation of Vγ7^+^ T-IEL.^36^ They also identified the human equivalent molecules, BTNL3/BTNL8 dimers on enterocytes, to be a similar selection molecule for Vγ4-expressing T-IEL in the human gut,^35–37^ thus providing the first evidence of intestinal epithelial expression of a TCR-selecting ligand, and of extrathymic selection and maturation of T-IEL. Intriguingly, continuous expression of BTNL3/8 seems to be required to maintain Vγ4^+^ T-IEL populations, as loss of BTNL3/8 expression in coeliac disease patients correlated with a loss of Vγ4^+^ T-IEL from the human gut.^38^

**Table 2:** Complete list of receptors known to be expressed by intestinal intraepithelial T lymphocytes, their ligands and their context-dependent functions.

Receptor	Alternative names	Ligands	Ligand expression on IEC	Function	Context
NKG2D	Klrk1 (gene)	MICA/MICB, ULBP (human) Rae-1, H60, MULT1 (mouse)	Induced^56^ Induced^56^	Costimulatory/stimulatory	In vitro killing assay^55,57,61^ Poly(I:C) treatment^58^ *Salmonella* and *E. moshkovskii* infection^59,60^ Coeliac disease^62–64^
CD160	By55	HVEM (high affinity) Classical and non-classical MHC-I molecules (low affinity)	Yes^74^ Yes	Costimulatory N/A	*Listeria monocytogenes* infection^75^ *C.rodentium* infection^74^
CD100	Sema4D	Plexins B1 and B2 CD72	Yes^76^ No^76^	Costimulatory	DSS-induced colitis (γδ T-IEL)^77^
CD8αα		Thymus leukemia antigen (TL)	Yes^84^	Inhibitory	In vitro costimulation^85,91^ Spontaneous model of T-cell-dependent colitis^89^
Ly49 Family		MHC-I molecules	Yes	Inhibitory No effect	In vitro costimulation^95^ DSS-TNBS-induced colitis and TNF^ΔARE^ ileitis model^99^ AOM-DSS-induced colorectal cancer^98^
CD200R	OX2R	CD200 iSEC1, iSEC2	No^102^ Yes^102^	Inhibitory	In vitro anti-CD3 stimulation^102^
CD94		HLA-E (human) Qa-1b (mouse)	Yes^124^ Yes^16^	Inhibitory (NKG2A dimerization) Costimulatory (NKG2C or E/H dimerization)	In vitro anti-CD3 stimulation^95,105^ in vitro anti-CD3 stimulation^105^ Coeliac disease^108,109^
2B4	SLAMF4 CD244.2	CD48 (SLAMF2)	N/A	Inhibitory	Ex vivo and in vivo anti-CD3 stimulation^95^
JAML	AMICA1	CAR	Yes^117,118^	Costimulatory	In vitro costimulation (γδ T-IEL only)^117^
OX40	CD134 TNFRSF4	OX40L (gp34, CD252, TNFSF4)	No	Costimulatory	In vitro anti-CD3 and anti-OX-40 stimulation^119^
NKp46	CD335 NCR1	Various viral and bacterial proteins		Stimulatory	In vitro cytotoxic assay^108,120^
NKp44	CD336 NCR2	Various viral and bacterial proteins		N/A	N/A
LAG-3	CD223	MHC-II molecules FGL1	Induced^125,126^ N/A	N/A	N/A
NKR-P1a	CD161 KLRB1	LLT1/CLEC2D (h)	Yes^127^	N/A	N/A
4-1BB	CD137 TNFRSF9	4-1BBL (CD137L, TNFSF9)	N/A	N/A	N/A
Gp49	LILRB4 ILT3 CD85k	Unknown	N/A	N/A	N/A
TIGIT	VSig9, Vstm3, WUCAM	CD155 CD112	Yes^128^ N/A	N/A	N/A
PD-1	CD279	PD-L1	Induced^129^	N/A	N/A
CTLA-4	CD152	B7.1/2	Yes^130^	N/A	N/A

*N/A* no data available, *IEC* intestinal epithelial cells, T-*IEL* intraepithelial T lymphocytes, *DSS* dextran sodium sulfate, *TNBS* trinitrobenzenesulfonic-acid, *AOM* azoxymethane.

With regards to innate-like αβ T-IEL, much progress has been made in exploring the selection pressures that drive specific TCR usage. Mayans et al.^39^ cloned the TCRs from DN (i.e. CD8αα) T-IEL and expressed these retrogenically in bone marrow (BM) to address whether the TCR is important for T-IEL selection. McDonald et al.^40^ also cloned αβ TCRs from adaptive and innate-like intestinal T-IEL populations and expressed them conditionally at the CD4^+^CD8^+^ double positive stage of thymic development in a mixed BM chimera system. Consistently, unconventional TCRs lead to the development of natural T-IEL, and conventional TCRs lead to the development of splenic CD8^+^ T cells or induced CD8^+^ αβ T-IEL. Together, both papers showed that strong agonist selection through the TCR drives the development of DN T-IEL from the thymus with further maturation taking place in the gut environment. Further, the cloned TCRs were not restricted on conventional major histocompatibility complex-1 (MHC-I), but were still mostly β2 microglobulin (β2m)-dependent, indicating their recognition of non-classical MHC-I, although some seemed to be cross-reactive, including one TCR that was MHC-II restricted. Indeed, the pool of negatively selected thymocytes from which T-IEL precursors develop express TCRs that are inherently cross-reactive to both MHC-I and MHC-II,^41^ which may be an important factor in the downregulation of both CD4 and CD8αβ. This was corroborated by another detailed study of CD8αα T-IEL precursors.^42^ This study also identified a distinct precursor population of CD8αα T-IEL, that more closely resembled iNKT cells, and was restricted on non-classical MHC-I and CD1d.

## Role of the TCR in T-IEL activation

The TCR is essential to activate functional responses in conventional T cells, and even for survival of some T cell subsets.^43–45^ In contrast, we do not know if T-IEL need functional TCRs and the TCR signal transduction machinery in order to get activated. Innate-like T cells are so called because of their ability to respond to cytokines and activating NK receptor ligands in the absence of TCR ligands. So, for example, CD27^-ve^ γδ T cells respond to the cytokines IL-1β and IL-23 to produce IL-17A,^46^ DETC respond to activation through the natural killer group 2D (NKG2D) receptor,^47^ and MAIT cells respond to IL-12 and IL-18 to produce Interferon γ (IFNγ).^48^

Notably, some innate-like T cell populations appear to have developmentally switched off TCR signaling. Thus, although thymic DETC progenitors are able to mobilize calcium in response to agonistic antibodies to the TCR, DETC isolated from the skin are no longer able to respond to TCR stimulation.^49^ Likewise, the TCR on both αβ and γδ innate-like intestinal T-IEL has been shown to be refractory to anti-CD3 mediated TCR triggering.^49,50^ Surprisingly, in vivo, the DETC TCR is constitutively phosphorylated and its downstream kinase Zap70 is enriched in epithelial contact zones of DETC.^51^ The putative DETC TCR ligand, Skint1, is constitutively expressed in the skin, providing a mechanism for constant TCR triggering in vivo that may be required for maintaining DETC. However, DETCs did not require a key TCR signaling intermediate, the Linker for Activation of T cell (LAT), for their long-term survival and homeostasis.^52^ Conversely, LAT was required for the DETC response to wounding of the skin,^52^ and DETC TCR tetramers bound to damaged keratinocytes even in mice lacking Skint1,^53^ implying the presence of a stress-induced TCR ligand that drives TCR activation for the immunosurveillance function of DETC.

In the context of γδ T-IEL, it is significant that Btnl1 is also constitutively expressed in the gut and that Vγ7^+^ T-IEL from *Btnl1*^−/−^ mice are more responsive to TCR agonistic antibodies than the corresponding cells from *Btnl1*^+/+^ mice.^35^ These data imply that constant Vγ7 TCR engagement by Btnl1 may be responsible for downmodulating TCR responsiveness. Primary Vγ7^+^ T-IEL co-cultured with MODE-K cells expressing Btnl1/Btnl6 dimers upregulated CD25, CD71, and Nur77-GFP, indicators of active TCR signaling.^36^ These responses were blocked by PP2, a pan-Src kinase inhibitor, supporting the notion that TCR engagement of Btnl molecules triggers TCR signaling. The human BTNL3/8-responding T-IEL express Vγ4, and through sequence analyses and comparisons with murine Vγ7, a critical interaction site with BTNL3/8 could be identified in framework region 3 (also known as hypervariable region 4) in the variable region of the gamma chain for both human Vγ4 and mouse Vγ7. Indeed, the binding of BTNL3 to human Vγ4 TCR was recently confirmed using surface plasmon resonance and other binding studies.^37^ This allowed the authors to make molecular modeling-based predictions about the binding geometry of Btnls to the TCRs. The data indicated that Btnls most likely bind in a non-conventional side-on conformation, akin to superantigens, molecules that can activate T cells independent of their TCR specificity. This also implies that Btnl binding to the T-IEL TCR still leaves the variable regions of the TCR free to bind to another ligand in the gut, that could be induced similar to the putative DETC TCR ligand mentioned above. However, the recent study by Willcox et al.^37^ indicates that it is unlikely that the TCR engages another ligand and a Btnl molecule simultaneously.

With the identification of TCR ligands for γδ T-IEL, and the indication of broad MHC reactivity of the TCRs on TCRαβ CD8αα T-IEL, we can begin to make predictions about the functions of the TCR on T-IEL. The data above indicate that innate-like T-IEL need the TCR for selection, and potentially expansion and retention in the gut. However, BTNL ligands appear to be downregulated in the context of both chronic inflammatory diseases and colorectal cancer^38,54^ suggesting that they do not activate IEL function in the gut. Additionally, both classical and non-classical MHC molecules can be constitutively and inducibly expressed by epithelial cells, however, it is unknown whether the endogenous, strong agonistic TCR ligands that select innate-like αβ T-IEL are expressed by IEC. Thus, it would appear contradictory to assume that stimulation of the TCR is needed for activation. Indeed, blocking TCR signaling using inhibitors against the TCR proximal kinases Zap70/Syk, or blocking antibodies, did not prevent infection-induced changes in γδ T-IEL migration and defense.^20^ Given that agonistic TCR antibodies do not induce signaling in both αβ and γδ innate-like T-IEL,^49,50^ other mechanisms must be in place to activate T-IEL. Below we present other receptors that may drive T-IEL activation, either directly, or, in a manner analogous to exhausted or antigen-experienced cells, by removing the inhibitory signals on T-IEL.

## Other (co) stimulatory receptors

A characteristic shared by innate-like T cells is that they express, in addition to the TCR, a wide range of activating and inhibitory NK receptors, indicating possible TCR-independent activation mechanisms. In the intestine, expression of such receptors is not limited to innate-like T-IEL as both human and murine induced T-IEL express NKG2D and CD94. In the following sections, in addition to reviewing data on the role of those receptors in innate-like T-IEL, we will also comment on evidence showing that induced T-IEL may undergo innate-like activation through NK receptors, specifically in the context of disease (Fig. 1 and Table 2).

## Natural killer group 2D

NKG2D was one of the first receptors identified as an activator of human αβ T-IEL cytotoxicity.^55^ NKG2D is a type II transmembrane C-type lectin activating receptor. It recognizes ligands upregulated by transformation, infection or cell stress, such as MICA/MICB and ULBP family members in humans, and Rae1, H60, and MULT1 in mice (Fig. 1). In humans, NKG2D is found at the surface of both resting and activated CD8^+^ T cells while murine T cells only express NKG2D when activated.^56^ Consistent with this, NKG2D is found at the surface of all murine DETC^57^ and human intestinal T-IEL, albeit at low levels.^55^ Although NKG2D is not expressed at the surface of murine intestinal T-IEL in normal conditions, cytokine-dependent NKG2D upregulation has been observed during infections. Hence, IL-15 mediates NKG2D expression on CD8αα T-IEL following poly(I:C) treatment in vivo,^58^ infection with *Entamoeba moshkovskii* triggers IFNγ production which is required for NKG2D expression on TCRαβ CD8αα T-IEL,^59^ and increased IL-1β production regulates γδ T-IEL NKG2D expression following *Salmonella typhimurium* infection in TLR9^−/−^ mice.^60^ Of note, in addition to upregulating NKG2D expression, these cytokines also facilitated NKG2D ligand expression on epithelial cells.^59,60^

NKG2D engagement triggers cytotoxicity of NK cells and T-IEL. Human intestinal CD8αβ T-IEL spontaneously kill selected colon cancer cell lines that express even low levels of NKG2D ligands, by triggering FasL-mediated cytotoxicity.^61^ However, freshly isolated human T-IEL did not kill other cell lines when triggered with anti-NKG2D antibodies,^55^ suggesting that colon cancer cell lines express other factors that enhance their killing. Murine poly(I:C)-treated intestinal T-IEL kill primary poly(I:C)-treated IEC in vitro and in vivo dependent on NKG2D/Rae-1 interactions.^58^ Infection of mice with *Salmonella*^16^ and *E. moshkovskii*^59^ also triggers NKG2D-mediated cytotoxicity of T-IEL against epithelial cells. NKG2D also functions as a potent costimulator of TCR-induced IFNγ production and proliferation of human intestinal T-IEL.^55^

Although human intestinal induced T-IEL constitutively express NKG2D, its expression can be further upregulated by IL-15.^55^ In coeliac disease, NKG2D-mediated IEL cytotoxicity is thought to be responsible for epithelial destruction.^62,63^ In the disease context, the chronic heightened expression of IL-15 by stressed IEC plays a critical role as IL-15 not only upregulates NKG2D expression on T-IEL, but also primes the cells and potentiates their cytotoxicity.^63^ NKG2D-mediated cytotoxicity depends on the phosphorylation of c-Jun N-terminal Kinase (JNK) and the activation of Extracellular signal-Regulated Kinase (ERK) by Phosphoinositide 3 kinase (PI3K)^62–64^ and the subsequent activation of cytosolic Phospholipase A2 (cPLA_2_)^64^ (Fig. 1). Interestingly, in vitro stimulation of human intestinal T-IEL by IL-15 enhanced ERK phosphorylation by PI3K as well as JNK and cPLA_2_ activation, and without this pre-stimulation, triggering of NKG2D on T-IEL could not induce degranulation.^64^ All these studies highlight a significant stimulatory function of NKG2D on T-IEL; however, it would appear that in most cases, it is not possible to rule out the simultaneous engagement of other receptors on T-IEL, nor licensing by IL-15 or other factors to permit NKG2D-mediated cytotoxicity.

## CD160

CD160 (recognized by the monoclonal antibody BY55) is an IgV-like domain membrane protein that was first identified on intestinal IEL^65^ (Fig. 1). Following alternative splicing, CD160 is found at the surface of cells either as a transmembrane receptor or attached by a glycophosphatidylinositol (GPI)-anchor.^66^ CD160 is expressed by both human and murine NK, T cells, NKT, Innate lymphoid cells 1 (ILC1), and CD8^+^ T-IEL,^65,67–69^ but the transmembrane isoform is restricted to NK cells.^66^ CD160 binds, with a low affinity, to classical and non-classical (CD1d) MHC-I molecules^70–72^ and with high affinity to the herpesvirus entry mediator (HVEM).^73^

Expression of CD160 by immune cells is possibly tissue- and microenvironment-dependent since in the colon of mice, it is restricted to T-IEL, most particularly to the CD8αα subset.^74^ Since HVEM is expressed by IEC, this suggests a role for CD160/HVEM in epithelial-mediated T-IEL response against pathogens. In line with this, HVEM^−/−^ mice displayed reduced epithelial integrity and bacterial clearance upon *Citrobacter rodentium* infection, as compared to wild-type (WT) mice. Binding of CD160 to HVEM on epithelial cells triggers Signal Transducer and Activator of Transcription 3 (STAT3) activation and subsequent expression of genes involved in response against pathogens.^74^ As IEL appear to be the major expressers of CD160 in the gut, it was postulated that CD160 on IEL binds to HVEM on IEC to drive these effects.

Opposing results came from a recent study showing that loss of CD160 on T-IEL did not affect *C. rodentium* host defense.^75^ However, CD160 was important for *Listeria monocytogenes* clearance, as 7 days post infection, CD160^−/−^ mice had more bacteria in the colon than WT mice. The authors associated the higher burden of bacteria with impaired Granzyme B expression and cytokine production by CD8^+^ T cells in CD160-deficient mice. These results suggest that engagement of CD160 positively regulates T-IEL, highlighting a stimulatory role of CD160 on T-IEL.

## CD100

CD100, also known as Sema4D, is a type IV transmembrane semaphorin that is highly expressed on T cells, with enhanced expression following activation, and to a lesser extent on NK, B cells, and antigen presenting cells (APCs).^76^ It is also found at the surface of all colonic T-IEL.^77^ CD100 binds to Plexins B1 and B2, which have broad expression and CD72, present on the surface of B cells and dendritic cells (DCs)^76^ (Fig. 1).

CD100 is known to have a costimulatory role in T cells^78^ and in CD100-deficient mice peripheral T cells function is impaired.^79,80^ More recently, CD100 was shown to bind to plexin B2 on keratinocytes and IEC to enhance γδ T cell responses.^81^ In this context, CD100 deficiency, specifically on DETCs and not any other T cells, led to delayed epidermal wound repair due to an inability of DETC to make morphological changes necessary for an effective response to tissue damage. These CD100-mediated morphological changes in DETC were shown to be ERK-dependent.^81^ In acute Dextran sodium sulfate (DSS)-induced colitis, CD100-deficiency led to enhanced susceptibility to the disease and a failure to resolve intestinal damage. The effect was attributed to CD100^−/−^ γδ T-IEL being unable to produce Keratinocyte Growth Factor (KGF), an important growth factor in stimulating epithelial proliferation. However, KGF production could not be stimulated by CD100 cross-linking on γδ T-IEL in vitro, suggesting that CD100 regulates γδ T-IEL functions in concert with other signals. Interestingly, although only γδ T-IEL secrete KGF, both CD100^−/−^ colonic γδ and αβ T-IEL were shown to have defective proliferative capacity following DSS-induced colitis.^77^ However, plexin B2-CD100 interactions did not play a role in αβ T cell activation in vitro,^81^ suggesting that while CD100 is highly expressed on all T cells, it functions in a subset- and site-specific manner, perhaps depending on the type of ligand expressed.

## Inhibitory receptors

In addition to the expression of many (co)stimulatory receptors, murine T-IEL express various inhibitory receptors that are thought to curb spurious T-IEL activation (Fig. 2 and Table 2). They are the focus of this section.

## CD8${\mathbf{\alpha}}{\mathbf{\alpha}}$

A defining characteristic of murine innate-like intestinal T-IEL is their exclusive expression of the CD8αα homodimer. CD8αα expression on lymphocytes is regulated by the intestinal environment as conventional CD4^+^ T cells also upregulate CD8αα expression upon migration to the intestine.^82,83^ CD8αα binds with high affinity to the Thymus Leukemia Antigen (TL),^84–88^ a nonclassical MHC-I molecule, whose expression is restricted to murine IEC. Unlike the TCR co-receptor CD8αβ expressed on conventional lymphocytes, CD8αα engagement negatively regulates TCR activation.^89^ CD8αα/TL interaction is thought to restrain T-IEL homeostatic proliferation^90^ and survival,^85^ preventing aberrant T-IEL proliferation without interfering with their immune functions. It has also been reported that TL/CD8αα interaction modulates T-IEL production of cytokines such as IL-2 and IFNγ and curbs T-IEL cytotoxicity.^85,91^ Yet TL deficiency does not lead to spontaneous colitis/IBD nor does it affect the outcome of chemically-induced colitis in mice. Interestingly, in TCRα^−/−^ mice, which spontaneously develop IBD, colitis developed earlier and was more severe when TL was concurrently deleted. This was linked to an increased secretion of IL-4 by TL^−/−^ TCRββ T-IEL, not usually found in normal context.^91^ Nevertheless, the involvement of CD8αα in the disease development is still unclear as TCRββ T-IEL do not express CD8αα. It is possible that TCRγδ T-IEL, which express CD8αα, are involved in the disease development. Alternatively, TL binding may not be restricted to CD8αα (and vice versa), and thus TL and CD8αα may function independently of each other.

## Ly49 family and Ly49E

The Ly49 receptors are murine functional homologs of human killer cell immunoglobulin-like receptors (KIR), and function as receptors for MHC-I. They are homodimeric type II glycoproteins of the C-type lectin-like superfamily and can be inhibitory or activating (Fig. 2). Ly49 receptors are expressed on innate immune cells (NK, NKT, DC, neutrophils, and macrophages) as well as on CD8^+^ T cells and each of these cell type express a unique complement of Ly49 receptors.^92^

All IEL express Ly49 receptors but the different populations of T-IEL do not share the same level of Ly49 receptor expression. Instead, 40% of TCRαβ CD8αα T-IEL are positive for members of Ly49 family when stained with a mixture of antibodies targeting Ly49A/C E/F/G2 against 10% of TCRγδ CD8αα T-IEL and <2% of TCRαβ CD8αβ T-IEL. TCRαβ CD8αα T-IEL mostly express Ly49E and Ly49F, where the Ly49E^+^ and Ly49F^+^ populations are nonoverlapping, and TCRγδ CD8αα T-IEL express Ly49E.^93–95^ Ly49E receptor is also found on the surface of DETC.^96^

TCRαβ CD8αα T-IEL expressing Ly49 receptors are hypo-responsive to TCR triggering, despite the capacity of these receptors to functionally couple to the TCR in an MHC-I independent way. Sequestration of these receptors, however, enhanced TCR stimulation, suggesting Ly49 receptors suppress TCR-mediated activation of T-IEL.^95^ In line with Ly49 receptors regulating T-IEL activation, in vitro TCR-mediated activation of skin and intestinal TCRγδ T-IEL results in an upregulation of Ly49E expression due to de novo synthesis of Ly49E molecules. Induction of Ly49E expression might indicate a negative regulatory feedback loop as basal and de novo expression of *Ly49e* gene seem to be differentially initiated and regulated depending on the activation state of T-IEL.^97^

Comprehensive studies led by Leclercq and colleagues ruled out a functional role for Ly49E receptors in cancer or colitis immune responses in mice. By comparing tumor size and frequencies in a mouse model of familial intestinal cancer, *Apc*^*Min/+*^ Ly49E WT, and *Apc*^*Min/−*^ Ly49E KO, as well as in a model of Azoxymethane (AOM)-DSS-induced colorectal cancer, the authors showed that tumor development and progression was not affected by Ly49E deletion.^98^ The same group also showed that the severity of inflammation in DSS and TNBS-induced colitis and the TNF^ΔARE^ ileitis models was similar in Ly49E KO and littermates control mice, indicating that the development and progression of IBD is not influenced by Ly49E expression on T-IEL in the systems examined.^99^

## CD200 receptor 1 (CD200R1)

CD200R1 is one of a paired receptor family that comprises 5 members (CD200R1, CD200R2–5, or CD200R-like receptors). Unlike the other members of the family that are activating, CD200R1 is an inhibitory receptor. It has three tyrosine residues that transduce inhibitory signals following phosphorylation by recruiting the adapter protein downstream of tyrosine kinase 2 (Dok2) and activating Ras GTPase-activating protein (RasGAP).^100^ CD200R1 is expressed by most myeloid and lymphoid cells and has been mainly studied in myeloid-type cells where it seems to play an anti-inflammatory role.^101^ CD200R1 is highly expressed on intestinal T-IEL.^102^

So far, three CD200R1 ligands have been discovered in mice: CD200 and the intestinal secretory cell-expressed 1 and 2 (iSEC1 and iSEC2).^102^ CD200 is broadly expressed and the two recently described CD200R1 ligands, iSEC1 and 2, are only expressed on IEC of the secretory lineage. Kojima et al., showed that all T-IEL constitutively express CD200R1 and that both iSEC1 and CD200 bind to CD200R1 on T-IEL. Co-stimulation of IL-2 stimulated T-IEL with anti-CD3 and iSEC1 or CD200 negatively affected IFNγ and TNFα production and inhibited T-IEL cytotoxic activity against a murine colonic adenocarcinoma cell line.^102^ These results suggest that CD200R1 binding to its ligands negatively regulates T-IEL functions, similar to its function on DETC.^103^ Further studies are awaited to address if CD200R1 signaling impacts intestinal T-IEL function in vivo, and whether iSEC1 and iSEC2 are the main ligands driving CD200R1 activation.

## Context-dependent regulatory receptors

This section focuses on receptors, such as CD94 and 2B4 that have been shown to exert both activating and inhibitory functions (Figs. 1 and 2; Table 2).

## CD94

CD94 is a glycoprotein that associates with members of the NKG2 family to form heterodimeric receptors transducing either inhibitory or activating signals. CD94/NKG2 receptor pairs are classic NK cell receptors (NKR) found abundantly on NK cells where they bind to ubiquitously expressed non-classical MHC class I molecule, HLA-E in humans, and Qa-1b in mice (Figs. 1 and 2). CD94/NKG2A (inhibitory) and CD94/NKG2C, NKG2E/H (activating) receptor pairs are also prominently expressed by subsets of memory/effector CTL and T-IEL.^104,105^ In humans, ~30% of T-IEL express CD94 and most of these cells are TCRαβ CD8αβ^+^. Within these, ~40% express the inhibitory NKG2A receptor pair. In functional studies using T-IEL cell lines, it was shown that T-IEL expressing NKG2A, regardless of co-expression of activating NKG2 types, were capable of inhibiting TCR-mediated cytolysis, suggesting that the inhibitory phenotype is dominant^105^ (Fig. 2). Conversely, CD94 cross-linking of CD94/NKG2C or NKG2E/H-expressing T-IEL cell lines markedly enhanced TCR-mediated cytolysis (Fig. 1). Similar results have also been obtained in mice, except that in mice TCRαβ CD8αα T-IEL express higher levels of the inhibitory CD94/NK2GA receptor complex, as compared to other T-IEL subsets.^95^

In addition to TCR stimulation, the expression of CD94 and its respective receptor partners on some T cells subsets, has also been linked to exposure to IL-15.^105–107^ In line with this, Jabri et al.^104^ reported that, in vitro, IL-15 alone was as effective as TCR-stimulation in upregulating CD94 expression in human T-IEL but not in CD8^+^ peripheral blood lymphocytes (PBLs). In coeliac disease, where there is chronic heightened expression of IL-15 by stressed epithelial cells, increased expression of CD94 and the activating CD94/NKG2C heterodimer, with a concomitant decrease of NKG2A on T-IEL has been observed.^104,108^ CD8^+^ TCRγδ T-IEL that express NKG2A are also decreased in active coeliac disease and these cells can suppress the cytotoxic programming of TCRαβ T-IEL, dependent on CD94/NKG2A engagement and TGFβ production.^109^ This suggests CD94 inhibitory function is lost in coeliac disease. Stimulation assays showed that NKG2C engagement increased proliferation and cytokine production of human T-IEL isolated from coeliac disease patients. In conjunction with the drastic upregulation of the HLA-E on enterocytes in coeliac disease as compared to healthy individuals, these data suggest that selective upregulation of the CD94/NKG2C in place of inhibitory CD94/NKG2A may be a mechanism of tipping the balance toward effector function of T-IEL in disease (Table 2).

## 2B4

2B4 (also known as SLAMF4 and CD244) is a member of the signaling lymphocyte activation molecule (SLAM) family, part of the immunoglobulin (Ig) domain containing superfamily (Fig. 2), and has two isoforms, a short and a long, derived from alternate splicing. It can function as both an activating and inhibitory receptor, depending on which splice variant is expressed, the density and glycosylation of the receptor or the availability of the signaling adapters, SLAM-associated protein (SAP) and Ewing’s sarcoma-associated transcript 2 (EAT-2).^110^ Its ligand is CD48 (SLAMF2). 2B4 is expressed by NK cells, and small populations of other lymphoid cells including DETC. Its expression on DETC is associated with cell activation, proliferation, and cytokine secretion.^111^

Gut microbiota products and CD70 co-stimulation both induce 2B4 expression on T-IEL in mice.^112,113^ It is therefore not surprising that 2B4 is found on almost all murine and human CD8^+^ T-IEL, although 2B4 is expressed more highly on CD8αα compared to CD8αβ T-IEL.^112,114^ Using *Slamf4*^*−/−*^ mice, and anti-SLAMF4 antibodies in combination with anti-CD3 stimulation, O’Keeffe et al., showed that 2B4 depletion or blockade in the context of anti-CD3 injections in mice induced CD8αβ T-IEL proliferation, cytokine production and increased T-IEL cytotoxicity, leading to higher gut inflammation.^114^ Higher gut inflammation was also found in EAT-2-deficient mice compared to WT after anti-CD3 challenge. These results suggest a negative regulatory role of 2B4 in T-IEL, after TCR triggering, probably mediated through the EAT-2 adapter protein (Fig. 2 and Table 2). However, a recent study using *Slamf4*^*−/−*^ mice showed that 2B4 expression drove a stronger protective immune response to oral *Citrobacter* and *Listeria* infections, and this correlated with reduced cytokine production from *Slamf4*^*−/−*^ T-IEL compared with WT T-IEL in vivo.^113^ These two studies are yet to be reconciled, although it should be pointed out that the earlier study mainly relied on anti-CD3 stimulation, a non-physiological stimulus. However, as CD48 can also signal into intestinal cells, and different subsets of T-IEL may express different isoforms of 2B4 and/or the adapters, the context of 2B4 triggering and the cell types involved in the intestine may decide the outcome.

## Other regulatory receptors

T-IEL express other regulatory receptors whose functions have never been studied in depth or in vivo (Table 2). For example, expression of JAML, a member of the Junctional Adhesion Molecule (JAM) family,^115^ a group of adhesion receptors in the Immunoglobulin (Ig) superfamily (Fig. 1) that regulate cell–cell interactions and leukocyte transmigration,^116^ has been characterized as a costimulatory receptor on DETC and intestinal γδ T-IEL.^117^ The JAML ligand was identified as the Coxsackie and Adenovirus Receptor (CAR), a member of the JAM family expressed on the surface of keratinocytes and IEC.^117,118^ In epidermal and intestinal γδ T cells, in vitro ligation of JAML in conjunction with TCR stimulation led to enhanced proliferation and production of the cytokines IL-2, Tumor Necrosis Factor-α (TNFα) and IFNγ as compared to TCR stimulation alone.^117^ Surprisingly, although JAML was expressed on both γδ and αβ T-IEL, anti-JAML antibodies only served to costimulate proliferation of γδ T-IEL. As JAML function in T-IEL has only been studied in vitro, it is still unclear whether JAML stimulation contributes to the functions of intestinal T-IEL in an in vivo setting.

Expression of another T cell costimulator, OX40 (also known as CD134 and TNFRSF4) is induced on all murine CD8^+^ T-IEL by in vitro anti-CD3 stimulation.^119^ Its expression is associated with IFN-γ production and enhanced T-IEL cytotoxicity. In vitro co-stimulation of T-IEL with anti-CD3 and anti-OX40 resulted in decreased IL-10 production compared with anti-CD3 stimulation alone (Table 2). Interestingly, while OX40L is not generally expressed on IEC or by T cells, anti-CD3 stimulation induced expression of OX40L on T-IEL, suggesting T-IEL potentially interact with each other via OX40 and OX40L. However, the relevance of this interaction has not been tested in vivo, and may be an artefact of in vitro culture systems.

In humans, a population of colonic CD8αβ^+^ γδ T-IEL that express Vδ1 constitutively express high levels of NKp46,^120^ a member of the natural cytotoxicity receptors family (NCR), normally found on NK cells.^121^ In vitro cytotoxic assays against K562 and colon adenocarcinoma cell lines showed that NKp46 expression on Vδ1 T-IEL is associated with high cytotoxic potential and IFNγ production. NKp46 expression is modulated by cytokines, as IL-2 and IL-15 upregulate the receptor expression on Vδ1 thymocytes.^120^ In line with this, NKp46 was also found on human CD8αβ^+^ αβ T-IEL in patients with coeliac disease. Similar to NKp46^+^ Vδ1^+^ T-IEL, T-IEL cell lines derived from those patients showed increased cytotoxicity and cytokine production.^108^ Both studies also found expression of NKp44 on human T-IEL (Table 2). These studies suggest that NCR expression on T-IEL is associated with an activated, effector phenotype.

In addition to the regulatory receptors mentioned, gene expression studies revealed that T-IEL express many other costimulatory and inhibitory receptors. Murine T-IEL express mRNA for LAG-3, NKR-P1A, 4-1BB, gp49/LILRB4, TIGIT, PD-1, and CTLA-4^93,94^ (Table 2). Protein level expression of LAG-3,^10,94^ TIGIT,^35^ and Gp49^112^ on CD8αα T-IEL has been confirmed by flow cytometry, while CTLA-4 is only expressed on CD8αβ^+^ αβ T-IEL.^10^ Although the functions of these receptors on other adaptive CD4^+^ and CD8^+^ T cells, and on intestinal T cells is well described and has been reviewed recently,^122^ to our knowledge, their role on T-IEL has never been studied.

## Conclusion and perspectives

In this review, we have focused on exciting advances on the TCR specificities of innate-like T-IEL, and on the plethora of novel signaling receptors that T-IEL express. Although the function of some of these is beginning to be unraveled in induced and innate-like T cells, their role in T-IEL activation and function is still unclear. Thus, the fundamental question of how T-IEL are activated remains unanswered. As multiple studies have shown, T-IEL are the primary immune responders to enteric infection and therefore must have a rapid response mechanism. On the other hand, exacerbation of their activation contributes to immune-mediated disorders such as IBD and coeliac disease. Understanding what drives T-IEL activation and what keeps them under control is crucial to developing therapeutic strategies against those diseases.

Although TCR expression is now fairly well-established to be necessary for agonist selection of αβ T-IEL in the thymus, and for selection of γδ T-IEL in the intestine, whether TCR engagement is necessary to fully activate T-IEL is still uncertain. To some extent, the T-IEL TCR is functional, as anti-CD3 T-IEL stimulation induces cytokine production and enhances T-IEL cytotoxicity. However, signaling networks in IEL need elucidation as TCR triggering does not lead to calcium release or phosphorylation of known downstream signaling effectors.^50^ The fact that T-IEL constitutively express so many costimulatory receptors suggest that T-IEL probably require more than one signal to be fully activated. Importantly, many of the functional studies on costimulatory receptors have relied on anti-CD3 costimulation, making it difficult to be certain about the physiological relevance of these studies. The activation signals may also be context-dependent, which makes the task of understanding how T-IEL get activated even more complex.

In summary, two possibilities for TCR-driven activation of T-IEL still remain - either the TCR on innate-like T-IEL is constitutively engaged, and release from inhibitory signals are necessary to trigger T-IEL activation, or there are as yet unknown TCR ligands that are upregulated upon stress, and these, together with costimulatory receptors, trigger activation of T-IEL. A third TCR-independent possibility is that the TCR on T-IEL is only required for the selection and retention of T-IEL in the intraepithelial space, and cytokines, together with activating receptors such as NKG2D trigger T-IEL activation, completely independent of TCR engagement. Notably, in our review of the literature, we did not find any evidence of activating receptors triggering T-IEL activation without prior exposure of T-IEL to cytokines and/or TCR stimulation. The challenge in the future will be to evaluate how T-IEL integrate information from multiple receptors to trigger an effector response. New mouse models that allow conditional deletion of coreceptors on T-IEL, in vitro T-IEL:epithelial coculture systems, such as those involving organoids^123^ and phosphoproteomic studies may help to provide new insights into the mechanisms driving T-IEL activation.
